# Effects of Vitamin E and Zinc on Growth Performance, Antioxidant Status, and Innate Immunity of Grass Carp (*Ctenopharyngodon idellus*)

**DOI:** 10.1155/anu/4049350

**Published:** 2026-07-22

**Authors:** Yao Cheng, Zhixiong Xu, Wenqiang Sun, Jianbing Wei, Shuihong Lv, Weifeng Li

**Affiliations:** ^1^ Pinglu Canal and Beibu Gulf Coastal Ecosystem Observation and Research Station of Guangxi, Guangxi Key Laboratory of Marine Environmental Disaster Processes and Ecological Protection Technology, College of Marine Sciences, Beibu Gulf University, Qinzhou 535011, China, en.bbgu.edu.cn

**Keywords:** antioxidant, co-supplementation, grass carp, immune response, vitamin E, zinc

## Abstract

An 8‐week feeding trial was conducted to investigate the effects of dietary co‐supplementation vitamin E (VE) and zinc (Zn) on growth performance, antioxidant status, and immune response of juvenile grass carp (*Ctenopharyngodon idellus*). The experimental design consisted of seven treatments with varying combinations of dietary VE and Zn levels (mg/kg): D‐0 (0:0), D‐1 (0:50), D‐2 (250:0), D‐3 (250:50), D‐4 (200:40), D‐5 (150:30), and D‐6 (100:20). A total of 5250 fish (average initial weight 228.11 ± 4.97 g) were randomly assigned into 21 cages (three replicates per group) and fed their respective diets twice daily for 56 days. Growth parameters, body composition, antioxidant activity, lysozyme, and complement C3 concentrations were analyzed. Significantly higher (*p* < 0.05) weight gain, specific growth rate, and feed efficiency were observed in the D‐4 group compared to the control and singly supplemented groups. Co‐supplementation also significantly enhanced serum, muscle, and liver SOD and T‐AOC activities and reduced MDA concentrations. The combined supplementation of 200 mg/kg VE and 40 mg/kg Zn showed resulting in the best overall performance under the current conditions. Although the independent or interaction effects of VE and Zn were not evaluated due to the limitations of fractional factorial design, these findings not only provide a basis for improving the immune ability of grass carp by reasonably co‐supplementation of VE and Zn, but also serve as a reference for reducing feeding costs in practical production.

## 1. Introduction

In 2024, the annual production of grass carp (*Ctenopharyngodon idellus*) exceeded 6.16 million tons, making it the most widely farmed and highest ‐producing freshwater fish in China. Grass carp is highly favored for its tender meat, delicious taste, low bone content, and high nutritional value [1, 2]. However, in recent years, high mortality rates in grass carp farming have been a persistent issue. Improving antioxidant capacity (AOC) and immunity is key to reducing grass carp mortality [2]. Currently, immunity enhancement through nutritional modulation has gained increasing recognition as understanding the nutritional requirements, feed selection, and feeding techniques provide important information about the physiological and nutritional requirements of fish.

Zinc (Zn) and vitamin E (VE) are important micronutrients that affect the physiology, immunity, and growth of fish [3]. On the one hand, Zn is an essential trace element for aquatic animals, serving as a critical component of over 200 enzymes, and supports strong antioxidant properties by eliminating free radicals on cell membranes [4], maintaining bio‐membrane structure, and enhancing immune function. Moreover, it is also essential for the development, differentiation, and function of various immune cells. Studies have confirmed that appropriate levels of Zn supplementation improves growth performance and AOC, such as percent weight gain and superoxide dismutase (SOD) activity in fish [3].

VE is a fat‐soluble vitamin that has multiple functions such as antioxidant, cell membrane protection, and immune function regulation. Aquatic animals cannot synthesize VE endogenously and therefore must obtain it from dietary sources. Optimal VE supplementation enhances the growth performance and muscle quality of mid‐growth grass carp [1], whereas long‐term VE deficiency impairs blood glucose metabolism, causing weight loss, delayed gonadal maturation, etc. In addition to growth and reproduction, VE also enhances AOC in fish by enhancing serum lysozyme activity, total complement activity, SOD, IgM contents, and complementing C3 content levels [3, 5–7]. Many studies have investigated the effects of adding VE or Zn supplements alone on fish growth, AOC, and immunity [8–12]. However, studies investigating the effects of VE and Zn in fish, particularly in grass carp, are scarce, especially in growth performance, antioxidant status, and immune response. Further research is needed to determine whether the co‐supplementation of VE and Zn may exhibit an interaction effect on enhancing the antioxidant defense system and immune response of grass carp, exceeding the effects of either nutrient alone.

The oxidative and stress indicators of fish not only reflect the health status of fish but also the antioxidant enzyme activity in the liver can serve as the main indicators of environmental pollution occurrence [13]. Improving the antioxidant and stress resistance of fish is crucial, as it not only helps to maintain their health but also promotes growth rate and enhances meat quality and yield. In this context, the present study aimed to evaluate the effects of dietary co‐supplementation with different levels of Zn and VE on growth performance and immunity (antioxidant enzyme activity, complement C3 content, and lysozyme activity) in grass carp. This study also seeks to identify the most effective dietary treatment that achieves the highest biological benefit under practical aquaculture conditions. The findings will provide scientific evidence for the nutritional optimization of herbivorous fish diets and offer valuable insights for enhancing aquaculture sustainability.

## 2. Materials and Methods

### 2.1. Experimental Fish and Trial Diets

A total of 5250 healthy juvenile grass carp (*Ctenopharyngodon idellus*) with an initial average body weight of 228.11 ± 4.97 g were selected before the experiment. The fish were randomly divided into seven treatment groups (D‐0 to D‐6) with three replicates per group (750 per group).

The diets were formulated with different levels of Zn and VE (mg kg^−1^), as shown in Table 1. All diets were iso‐nitrogenous and iso‐lipidic, with fishmeal, soybean meal, and casein as the primary protein sources. VE (all‐rac‐α‐tocopheryl acetate, ≥96%) and Zn (zinc sulfate monohydrate, analytical grade) were purchased from Sigma–Aldrich. The experimental design was based on Ding et al. [14] and modified from Pohlenz et al. [15] and Cheng et al. [16]. The additional quantities of VE [17] and Zn [18] were based on the references, respectively.

**Table 1:** Experimental diet formulation with different levels of Zn and VE inclusion (%, dry matter).

Ingredient	D‐0	D‐1	D‐2	D‐3	D‐4	D‐5	D‐6
Fish meal^a^	17.00	17.00	17.00	17.00	17.00	17.00	17.00
Soybean meal	20.35	20.35	20.35	20.35	20.35	20.35	20.35
Casein^b^	10.00	10.00	10.00	10.00	10.00	10.00	10.00
Wheat meal	20.00	20.00	20.00	20.00	20.00	20.00	20.00
Fish oil^a^	2.00	2.00	2.00	2.00	2.00	2.00	2.00
Soybean oil	2.00	2.00	2.00	2.00	2.00	2.00	2.00
Vitamin premix (vitamin E free)^c^	1.00	1.00	1.00	1.00	1.00	1.00	1.00
Mineral premix^d^	2.00	2.00	2.00	2.00	2.00	2.00	2.00
Cellulose	22.00	21.995	21.975	21.97	21.976	21.982	21.988
Monocalcium phosphate (MCP)	2.00	2.00	2.00	2.00	2.00	2.00	2.00
Choline chloride (50%)	0.60	0.60	0.60	0.60	0.60	0.60	0.60
Ethoxyquin (30%)	0.05	0.05	0.05	0.05	0.05	0.05	0.05
Carboxymethylcellulose sodium	1.00	1.00	1.00	1.00	1.00	1.00	1.00
Vitamin E (mg/kg)	0.00	0.00	250.00	250.00	200.00	150.00	100.00
Zinc (mg/kg)	0.00	50.00	0.00	50.00	40.00	30.00	20.00
Proximate composition (%)							
Moisture	11.60	11.70	11.75	11.68	11.54	11.73	11.68
Crude protein	31.43	31.52	31.47	31.32	31.54	31.37	31.48
Crude fat	5.56	5.59	5.57	5.56	5.57	5.54	5.55
Ash	13.82	13.73	13.80	13.70	13.77	13.64	13.76
Vitamin E (mg/kg)	4.20	3.90	255.30	254.00	206.70	153.20	100.40
Zinc (mg/kg)	15.00	64.70	13.80	64.50	55.10	44.60	35.40

^a^North Pacific white fishmeal and fish oil (EPA + DHA ≥20%) from American Seafood Company, USA.

^b^Casein, human food grade, was bought from Guangzhou Branch of Gansu Hualing Milk Company, China.

^c^Vitamin premix (per kg of diet): vitamin A, 2000 IU; vitamin B_1_ (thiamin), 5 mg; vitamin B_2_ (riboflavin), 5 mg; vitamin B_6_, 5 mg; vitamin B_12_, 0.025 mg; vitamin D_3_, 1200 IU; vitamin K_3_, 2.5 mg; folic acid, 1.3 mg; biotin, 0.05 mg; pantothenic acid calcium, 20 mg; inositol, 60 mg; ascorbic acid (35%), 110 mg; niacinamide, 25 mg.

^d^Mineral premix (per kg of diet): MnSO_4_, 10 mg; MgSO_4_, 10 mg; KCl, 95 mg; NaCl, 165 mg; KI, 1 mg; CuSO_4_, 12.5 mg; FeSO_4_, 105 mg; Na_2_SeO_3_, 0.1 mg; Co, 1.5 mg.

### 2.2. Experiment and Sampling

The experiment was conducted in net cages at Fengtinghe Reservoir in Shangsi County, Fangchenggang City, Guangxi. Prior to fish acquisition, the ponds were thoroughly cleaned and disinfected. After procurement, the fish underwent a 2‐week acclimatization period before being weighed and grouped for the formal experiment. The grass carp were randomly reared in net cages measuring 4.0 m × 2.0 m, totaling 21 cages and 250 fish per net cage. Each cage was placed in the reservoir, where the water Zn content was below 0.02 mg/L.

The trial lasted 56 days, with feeding conducted twice daily (8:00–10:00 and 15:00–17:00). Feed trays were placed at the bottom of the cages, and the feeding rate was adjusted to ensure slight leftovers 30 min post‐feeding. The leftover feed was collected daily, sun‐dried, and weighed to record residual amounts accurately. Fish health was monitored weekly. Water temperature at 1.5 m depth averaged 28°C, while at 0.5 m depth, it ranged between 26°C and 31°C. The pH was 7.5 ± 0.5, and dissolved oxygen levels were 6.5–7.5 mg/L.

After a 24 h fasting period at the end of the trial, three fish per replicate cage were randomly sampled, anesthetized with MS‐222 (tricaine methanesulfonate, 100 mg/L, buffered with sodium bicarbonate), total length, body length, and body weight were measured and recorded, and used for blood and tissue sampling. Surface moisture was removed using a gauze. Blood was drawn from the caudal vein using a heparinized (0.2% sodium heparin) syringe and centrifuged at 3000 rpm for 15 min to obtain serum, which was aliquoted into three 2 mL centrifuge tubes and stored at −80°C in liquid nitrogen for enzyme activity assays. Liver, spleen, head kidney, and muscle tissues were dissected, packaged, and stored at −80°C. Total weight and fish count per cage were recorded. Three fish per cage were preserved in an ultra‐low‐temperature freezer for whole‐body analysis.

### 2.3. Determination of Growth Performance Metrics

Survival rate, relative growth rate, specific growth rate, and feed efficiency were calculated as follows:

Survival rate SR,%: Final fish count/Initial fish count×100,

Relative growth rate RGR,%: Final weight−Initial weight/Initial weight×100,

Specific growth rate SGR,%/day: ln Final weight−ln Initial weight/Days×100,

Feed efficiency FE,%: Weight gain/Feed intake×100,

Condition factor CF: Body weight/Body length3×100.

### 2.4. Proximate Composition and Diet Analysis

The proximate composition (moisture, crude protein, crude lipid, and ash) of diets and whole fish was determined following AOAC (2005) [19] standard procedures.

Moisture: oven drying at 105°C.
Crude protein: Kjeldahl method (*N* × 6.25).
Crude lipid: Soxhlet extraction.
Ash: muffle furnace combustion at 550°C.

VE levels in diets were quantified via HPLC with UV detection (292 nm). The Zn content was analyzed using inductively coupled plasma–optical emission spectrometry (ICP‐OES).

### 2.5. Antioxidant and Immune Parameter Assays

Liver, spleen, head kidney, and muscle tissues were homogenized in ice‐cold physiological saline to obtain 10% and 1% homogenates for enzyme assays. The protein concentration was determined using the Bradford method.

The following biochemical parameters were measured using commercial assay kits (Nanjing Jiancheng Bioengineering Institute, China):

SOD: inhibition of pyrogallol autoxidation.
Total AOC (T‐AOC): ferric‐reducing antioxidant power.
Malondialdehyde (MDA): thiobarbituric acid reactive substances (TBARS).
Lysozyme: turbidimetric assay using Micrococcus lysodeikticus.
Complement C3: ELISA method.

### 2.6. Statistical Analysis

Data are presented as means ± standard deviation (SD). Statistical analyses were performed using SPSS 19.0.

An one‐way ANOVA was conducted to test the differences between each experimental group. When significant differences were found (*p* < 0.05), Duncan’s multiple range test was applied for post hoc comparisons. Although less conservative than Tukey’s test, Duncan’s method was chosen due to its suitability for detecting subtle differences in biological data. Before ANOVA, data were tested for normality (Shapiro–Wilk) and homogeneity of variances (Levene’s test).

The experimental unit for all statistical analyses was the cage (*n* = 3 replicates per treatment). Subsamples from the fish were pooled prior to analysis.

## 3. Results

### 3.1. Effects of Zn and VE on Growth Performance and Survival

**Figure 1:**
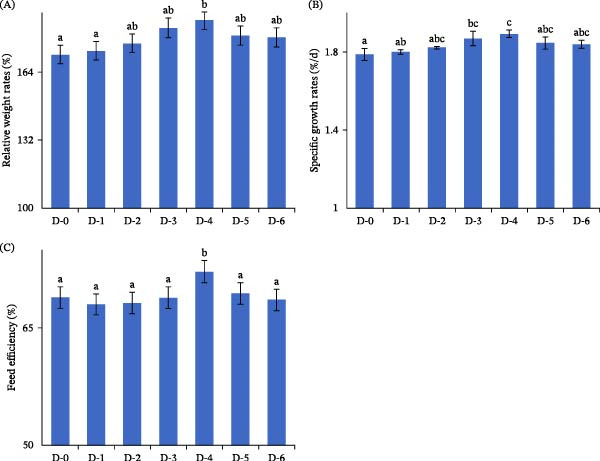
The effects of Zn and / or VE inclusion on the relative weight rate (A), specific growth rate (B), and feed efficiency (C) of grass carp. All above data are means ± SD (*n* = 3). Different letters indicate significant differences among groups (*p* < 0.05), while the same letter indicates no significant difference (*p* > 0.05).

The results of this study show no significant differences (*p* > 0.05) in survival rate (97.03%−97.89%) and CF (1.73–1.84) among groups. The effects of Zn and/or VE inclusion on the feed efficiency, relative weight rate, and specific growth rate of grass carp are illustrated in Figure 1. In general, the D‐4 group exhibited significantly higher (*p* < 0.05) relative weight rate and specific growth rate compared to the D‐0 and D‐1, whereas the feed efficiency of D‐4 group was significantly higher (*p* < 0.05) compared to other groups (Figure 1).

### 3.2. Effects on Whole‐Body Proximate Composition

The effects of Zn and/or VE inclusion on the proximate composition of grass carp are summarized in Table 2. In general, statistical analysis revealed significant differences (*p* < 0.05) in moisture, crude protein, crude lipid and ash among groups, though no consistent pattern was observed. For crude protein content, while no significant differences existed among most groups (*p* > 0.05). For ash content, the D‐6 group showed significant higher (*p* < 0.05), while D‐5 had the lowest (*p* < 0.05) ash than other groups.

**Table 2:** The effects of Zn and/or VE inclusion on the proximate composition of grass carp.

Diet	Moisture (%)	Crude protein (%)	Crude lipid (%)	Ash (%)
D‐0	72.63 ± 0.41^d^	13.22 ± 0.30^a^	10.22 ± 0.77^cd^	2.89 ± 0.13^a^
D‐1	72.69 ± 0.69^d^	13.49 ± 0.83^ab^	10.00 ± 0.61^bc^	3.02 ± 0.22^b^
D‐2	71.72 ± 0.88^bc^	13.67 ± 0.47^ab^	9.51 ± 1.49^ab^	3.02 ± 0.20^b^
D‐3	72.22 ± 0.41^cd^	13.93 ± 1.06^abc^	10.30 ± 0.52^cd^	2.88 ± 0.09^a^
D‐4	71.02 ± 0.44^ab^	14.62 ± 0.75^c^	9.31 ± 1.39^a^	3.08 ± 0.17^b^
D‐5	71.39 ± 1.54^ab^	14.26 ± 1.12^bc^	10.81 ± 1.09^d^	2.86 ± 0.16^a^
D‐6	70.80 ± 0.79^a^	14.01 ± 1.07^bc^	10.04 ± 0.25^bc^	3.11 ± 0.07^b^

*Note:* Values are presented as means ± SD (*n* = 3). Values with different superscripts in the same row are significantly (*p* < 0.05) different.

### 3.3. Effects on AOC

**Figure 2:**
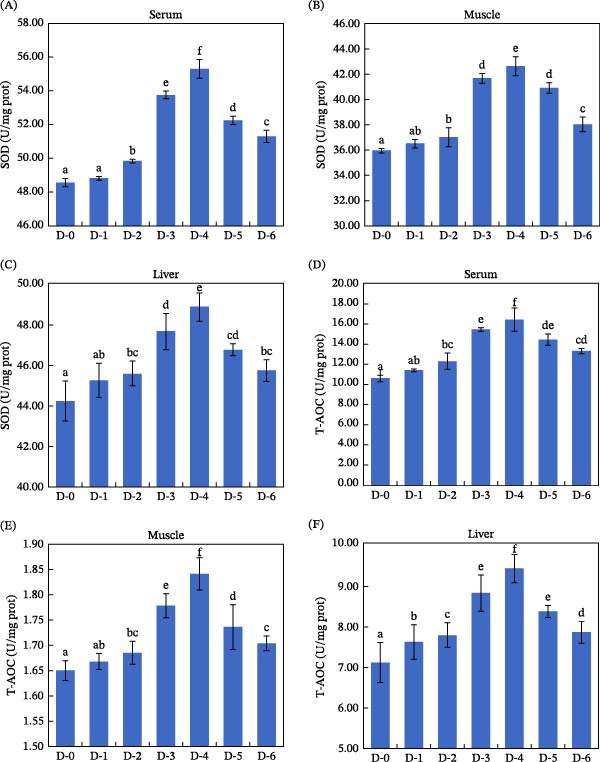
The effects of Zn and/or VE inclusion on the SOD and T‐AOC in serum (A and D), muscle (B and E), and liver (C and F) of grass carp. All above data are means ± SD (*n* = 3). Different superscript letters in the columns indicate significant difference (*p* < 0.05), while the same letter indicates no significant difference (*p* > 0.05).

Figure 2 shows the activities of SOD and T‐AOC in the serum, liver, and muscle tissues. Both indicators were highest in the D‐4 group across all tissues. Compared with D‐0, D‐1, and D‐2, the combined supplementation groups (D‐3 to D‐6) showed significantly enhanced SOD and T‐AOC activities (*p* < 0.05).

### 3.4. Effects on Lysozyme Activity and MDA Levels

**Figure 3:**
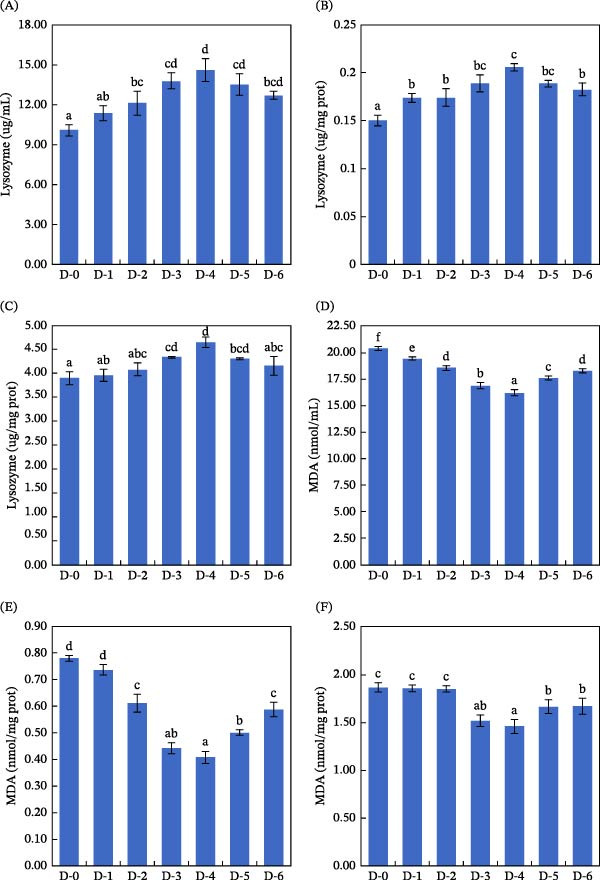
The effects of Zn and/or VE inclusion on the lysozyme activity in serum (A), spleen (B), and head kidney (C), and malonaldehyde content in serum (D), liver (E), and muscle (F) of grass carp. All above data are means ± SD (*n* = 3). Different superscript letters in the columns indicate significant difference (*p* < 0.05).

As shown in Figure 3, lysozyme activity in serum, spleen, and head kidney was significantly higher in group D‐4 compared to group D‐0, D‐1, and D‐2 (*p* < 0.05). Group D‐2 (VE only) showed greater serum lysozyme than D‐0, while D‐1 (Zn only) showed no significant difference from the control. In the spleen, both D‐1 and D‐2 showed improvement compared to D‐0, with the best responses in co‐supplemented groups.

MDA concentrations in serum, liver, and muscle followed a declining trend with increasing VE and Zn levels. The lowest MDA values were observed in group D‐4 (*p* < 0.05), indicating reduced oxidative damage.

### 3.5. Effects on Serum Complement C3 Concentration

**Figure 4:**
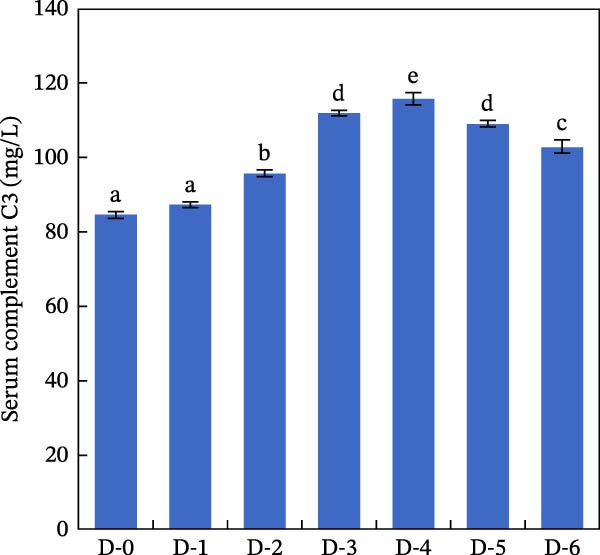
Effects of Zn and/or VE on serum complement C3 concentration in grass carp. Different superscript letters in the columns indicate significant difference (*p* < 0.05).

Complement C3 concentrations are presented in Figure 4. No significant change was observed between D‐1 and D‐0 (*p* > 0.05), while D‐2 (VE alone) was significantly higher than the control. Combined supplementation (D‐3 to D‐6) led to even greater C3 concentrations, with the highest levels found in D‐4 (*p* < 0.05), indicating the effect of Zn and VE on nonspecific immunity.

## 4. Discussion

This study evaluated the effects of seven dietary supplementation with Zn and VE, individually and in combination, on growth performance, AOC, and immune response in juvenile grass carp (228–657 g). The results demonstrated that co‐supplementation with Zn and VE at appropriate levels, particularly at 40 mg/kg Zn and 200 mg/kg VE (D‐4), significantly improved growth, antioxidant enzyme activity, and immune markers under the conditions of this experiment. However, due to the lack of full factor design in this study, the submitted data were limited for constructing a reliable regression model, and it is difficult to investigate interaction trends and evaluate optimal VE and Zn‐specific levels.

### 4.1. Growth Performance and Nutrient Synergy

Growth performance (RGR, SGR, and FE) was significantly enhanced in the co‐supplemented groups compared to the control and single‐nutrient treatments. D‐3 and D‐4 showed significantly higher relative weight rate and specific growth rate compared to D‐0, D‐1 (Zn alone), and D‐2 (VE alone); the highest growth performance was recorded in group D‐4, confirming that supplementing Zn and VE to enhance growth performance. These findings align with Wang et al. [20], Liu et al. [21], and Lv et al. [22], in which Zn supplementation improve the growth of groupers and yellow catfish, respectively. Similarly, VE supplementation has been shown to improve the growth performance of black carp [23] and hybrid red tilapia [24]. In this experiment, although some differences in the fish whole‐body proximate composition, there was no consistent pattern with supplementation of VE and Zn; this may be due to differences in fish body composition due to metabolic regulation rather than consistent trends observed in different treatments.

### 4.2. Antioxidant Defense Enhancement

The serum T‐AOC reflects the combined antioxidant effects of the body’s non‐enzymatic antioxidant system and antioxidant enzyme system [25]. SOD and T‐AOC activities in the serum, liver, and muscle were significantly higher in D‐3 to D‐6 groups, particularly D‐4, compared to both the D‐0 and D‐1 groups. These results align with known roles of Zn as a cofactor of Cu/Zn‐SOD and VE as a lipid‐soluble antioxidant that protects polyunsaturated fatty acids in cellular membranes. Previous studies have also reported that Zn and VE together improve antioxidant status and reduce oxidative stress in aquatic animals and mammals [9, 26–28]. In research on optimal dietary Zn levels for juvenile cobia, Zn supplementation significantly affected the Zn content in liver and other tissues [29]. The enhanced antioxidant enzyme activity in D‐4 may be attributed to the adequate provision of cofactors and membrane stabilizers that support the enzyme expression and activity. Furthermore, investigations into the impact of Zn and VE on oxidative stress and non‐enzymatic glycation in diabetic rats showed that both nutrients exert antioxidant effects by reducing free radical generation and create a reducing environment for monosaccharides (e.g., glucose), preventing autoxidation and blocking the initiation of nonenzymatic glycation. Combined Zn and VE supplementation proved more effective than individual supplementation [30], aligning with the results of this experiment.

Studies have shown that in turbot, hepatic T‐AOC increases significantly with higher dietary VE levels, suggesting that VE supplementation can enhance the body’s AOC and reduce oxidative stress [31]. In studies on the effects of Zn and VE on lipid peroxidation and glucose metabolism in rats with liver cirrhosis, VE reduced lipid peroxidation and mitigated acute liver injury caused by galactosamine. Patients with liver cirrhosis often have reduced hepatic Zn levels, and blood Zn levels vary with the degree of hepatocellular necrosis [32]. Zn, an essential trace nutrient, protects cells by controlling peroxidation; meanwhile, VE, as a frontline antioxidant, protects polyunsaturated fatty acids in cell membranes during early free radical attacks through its free radical scavenging role in biological membranes [33]. These findings confirm the antioxidant function of Zn and VE, which is similar to the results of this experiment.

### 4.3. Immune Response Modulation

Lysozyme is an important hydrolase enzyme that catalyzes the hydrolysis of bacterial cell walls, thereby killing pathogenic microorganisms. Measuring the lysozyme content in serum and tissues can be used as an indicator of nonspecific immune capacity in fish [30]. Numerous studies have shown that both Zn and VE can increase lysozyme levels, thereby enhancing the body’s immune function. Yang [34] found in common carp that serum lysozyme activity significantly decreased when VE was deficient, while supplementation with VE led to a significant increase in serum lysozyme levels. However, when VE was added in high doses, lysozyme activity decreased again, indicating that an appropriate level of VE enhances lysozyme activity in carp, whereas excessive VE has an inhibitory effect. Wu et al. [35] showed that Zn can increase lysozyme levels in mid‐growth stage grass carp, with the highest lysozyme content in the spleen and head kidney observed at a dietary Zn level of 59.17 mg/kg. Excessive Zn levels, however, led to a decrease in the lysozyme content. In the present study, D‐2 lysozyme levels in the spleen and serum of grass carp were significantly higher compared to the control group D‐0, consistent with the findings of Yang [34]. The D‐1 lysozyme content in the spleen was also significantly higher than D‐0, consistent with the results of Wu et al. [35]. When Zn and VE were added together, lysozyme levels in the serum, spleen, and head kidney all increased compared to D‐0 to D‐2 groups. The highest levels were observed in group D‐4, indicating that the best lysozyme activity was achieved at dietary levels of 40 mg of Zn/kg and 200 mg of VE/kg.

The complement system serves as a crucial bridge connecting the non‐specific and specific immune responses in fish and is central to many immune defense mechanisms. Complement component C3 is the core component of the complement system and a key hub in the complement activation pathways [36], playing an important role in the body’s nonspecific immune function. Studies have shown that an appropriate dietary Zn level can increase complement C3 levels in grass carp. The highest C3 levels in mid‐growth stage grass carp were observed at a dietary Zn concentration of 55.17 mg/kg, while excessive Zn levels led to a decrease in the C3 content [35]. Other research has indicated that VE can promote the synthesis of complement C3 protein [37]. However, the results of this experiment showed that when Zn was added at 50 mg/kg (D‐1), serum complement C3 levels did not significantly increase compared to those of D‐0, which is inconsistent with the findings of the above‐mentioned study. Interestingly, when Zn and VE were added together, serum C3 levels did not decrease but instead increased significantly. This suggests that an appropriate amount of Zn and VE can promote C3 production. Serum lysozyme and complement C3 concentrations, as markers of innate immunity, were significantly elevated in D‐3 to D‐6 groups. This suggests that Zn and VE not only protect cellular structures but also enhance humoral immune responses, although the exact molecular pathways warrant further investigation.

### 4.4. MDA Reduction and Oxidative Damage

MDA is a lipid peroxidation product generated during the metabolism of oxygen‐free radicals in the body. It is widely used as an indicator of oxidative damage to cell membranes [9]. Studies on yellow catfish [38] and hybrid red tilapia [24] have shown that VE can effectively reduce MDA levels in fish. In a study on *Cynoglossus semilaevis*, it was found that the lowest MDA levels in serum, liver, and spleen occurred when Zn was added at 18 mg/kg, in muscle at 54 mg/kg, and in the kidney at 96 mg/kg [39]. Wu et al. [35] found that in mid‐growth stage grass carp, MDA levels initially decreased and then plateaued as dietary Zn levels increased. Patrice et al. [40] reported a synergistic antioxidant effect of Zn and VE in diabetic mice. In the current experiment, D‐1 significantly reduced serum MDA levels compared to D‐0 but had no notable effect on MDA levels in muscle and liver, which contrasts with the findings of previous studies. This discrepancy may be due to differences in the test species, feed formulations, or experimental conditions. MDA levels in both the serum and liver in D‐2 were significantly reduced compared to D‐0, consistent with the findings of Chen et al. [38]. This indicates that Zn and VE can reduce serum MDA levels in grass carp, enhance AOC, and decrease oxidative damage to cell membranes, thereby boosting the immune function. Wang et al. [41], in a study on juvenile crabs, found that when VE was added at 100 mg/kg, MDA levels in the hepatopancreas were significantly lower than those in both the non‐supplemented group and the group receiving 300 mg/kg VE, suggesting that the antioxidant effect of VE is dose‐dependent. The current study showed that D‐3 to D‐5 significantly reduced MDA levels in the serum, liver, and muscle of grass carp, and D‐4 exhibited the lowest MDA levels, suggesting the most effective protection against oxidative damage.

### 4.5. Future Directions

This study used a linear gradient design rather than a complete factorial design, which limits the ability to fully dissect the independent effects and the interactive effects of VE and Zn across graded levels statistically. Also, certain physiological mechanisms, such as the gene expression of antioxidant enzymes or cytokines, were not investigated.

Future studies should apply transcriptomic or proteomic approaches to clarify the molecular pathways underlying Zn–VE synergy.

## 5. Conclusions

In summary, dietary co‐supplementation of VE and Zn significantly enhanced growth performance, AOC, and innate immune response in juvenile grass carp, with the combination of 200 mg/kg VE + 40 mg/kg Zn (D‐4) yielding the best overall results under the present experimental conditions. This effect likely results from the complementary roles of VE in membrane stabilization and free radical scavenging and Zn as a cofactor for antioxidant enzymes and immune mediators. Further research will use molecular analysis to elucidate the potential mechanism of the Zn–VE interaction effect.

## Author Contributions

**Yao Cheng:** investigation, methodology, software, writing – original draft. **Zhixiong Xu:** writing – review and editing, funding acquisition, supervision. **Wenqiang Sun**, **Jianbing Wei, and Shuihong Lv:** investigation, data curation, formal analysis. **Weifeng Li:** conceptualization, supervision, resources, funding acquisition, writing – review and editing.

## Funding

The present study was financially supported by the National Natural Science Foundation of China (32260920) and High‐level Talents Scientific Research Start‐Up Fund Project of Beibu Gulf University (2021KYQD08).

## Disclosure

This research will provide valuable insights into the metabolic mechanisms underlying the combined supplementation of vitamin E and zinc in fish. All authors have read and agreed to the published version of the manuscript.

## Ethics Statement

All experimental procedures used were conducted with the approval of the Animal Care Advisory Committee of Beibu Gulf University (Approval Code: LW2024‐1026, Approval Date: 2024‐01‐05).

## Consent

The authors have nothing to report.

## Conflicts of Interest

The authors declare no conflicts of interest.

## Data Availability

The data that support the findings of this study are available from the corresponding author upon reasonable request.
